# Health-Related Quality of Life, Body Mass Index and the 10-Metre Walk Test in Patients Awaiting Total Knee or Hip Arthroplasty: A Cross-Sectional Data Analysis with Matched Controls

**DOI:** 10.3390/life15020231

**Published:** 2025-02-05

**Authors:** Monika Pavlović, Špela Matko, Ferdinand Prüfer, Stefan Löfler, Michael J. Fischer, Vincent Grote, Nejc Šarabon

**Affiliations:** 1 Department of Prosthetics, Faculty of Health Sciences, University of Ljubljana, 1000 Ljubljana, Slovenia; 2 Department of Kinesiology, Faculty of Health Sciences, University of Primorska, 6310 Izola, Slovenia; 3 Ludwig Boltzmann Institute for Rehabilitation Research, 1140 Vienna, Austria; 4 Institute for Outcomes Research, Center for Medical Data Science, Medical University of Vienna, 1090 Vienna, Austria; 5 Physiko & Rheumatherapie, Institute for Physical Medicine and Rehabilitation, 3100 St. Pölten, Austria; 6 Rehabilitation Center Kitzbühel, 6370 Kitzbühel, Austria

**Keywords:** osteoarthritis, arthroplasty, knee replacement, hip replacement, health-related quality of life, body mass index, walking speed

## Abstract

The study compares the body mass index (BMI), 10-m walk test results, and self-rated health-related quality of life (HRQoL) of patients awaiting total knee or hip arthroplasty with age and sex-matched controls. Additionally, we investigated relationships between these variables to better understand how mobility impairments and HRQoL contribute to the need for surgical intervention. Forty-three patients (age: 66.7 ± 8.7 years) awaiting total knee arthroplasty (*n* = 23) or total hip arthroplasty (*n* = 20) and 54 healthy control individuals (age: 65.6 ± 1.5 years) participated in this study. Weight and height were measured, the BMI was calculated, the 10 m walk test was performed, and HRQoL was assessed using the EQ-5D-5L questionnaire. Patients had a significantly higher BMI than controls (*p* = 0.037), with the majority of both groups classified as overweight or obese (patients, 86%; controls, 73%). Patients also walked significantly more slowly than controls (*p* < 0.001). HRQoL was significantly lower in patients across all dimensions (*p* < 0.001), with the greatest impairments observed in mobility, usual activities, and pain. Significant but weak correlations (*p* = 0.001–0.042, *r_s_* = 0.31–0.48) were found between the HRQoL, BMI, and 10 m walk test results. These findings confirm that patients awaiting total knee or hip arthroplasty exhibit an increased BMI, reduced locomotor function, and impaired HRQoL, highlighting the extent of functional limitations in individuals with end-stage osteoarthritis. The strong association between mobility impairments and HRQoL further underscores the impact of osteoarthritis on daily life and the increasing need for surgical intervention.

## 1. Introduction

Osteoarthritis (OA) is a degenerative joint disease that affects one or more joints and is characterised by cartilage destruction and changes in the underlying bone. It is a complex condition influenced by genetic, biomechanical, and inflammatory factors, with age and obesity being among the most common risk factors [1,2]. Many studies have linked being overweight, indicated by an increased body mass index (BMI), to a higher risk of developing OA, particularly in weight-bearing joints [3]. OA affects one in three people over the age of 65, and women are more frequently affected than men [4]. The most common symptom is progressive pain in the affected joint, followed by stiffness and limited mobility [5]. With 12.6% of its residents diagnosed with hip OA, Europe has the highest prevalence of this disease worldwide [6]. The global incidence of knee OA was 203/10,000 patients/year aged 20 years or more. This means that approximately 86 million people (20 years and older) suffer from OA worldwide [7]. Consequently, OA is associated with global socioeconomic and health burdens [1,7].

Total knee arthroplasty (TKA) and total hip arthroplasty (THA) are common surgical techniques for the treatment of the functional limitations and discomfort/pain caused by OA [8]. The demand for these interventions continues to rise with the aging population and the increasing prevalence of OA. Thus, interest is steadily growing in understanding their impacts on various health outcomes, such as BMI, functional outcomes, and health-related quality of life (HRQoL) [9,10]. Previous studies have shown that BMI is a significant predictor of surgical outcomes and postoperative complications in joint arthroplasty patients [10,11]. In addition, locomotor function, as measured by the 10-m walk test (10MWT), is critical for assessing the success of TKA and THA procedures, as it reflects the patient’s ability to perform activities of daily living and navigate their postoperative environment [12]. Individuals with slower gait speeds face an increased likelihood of disability, cognitive impairment, institutionalisation, falls, and death [13]. In addition, HRQoL is an important outcome measure that captures the multidimensional impact of OA and its treatment on patients’ physical, emotional, and social well-being [14,15]. While postoperative improvements in HRQoL have been well documented, studies on preoperative conditions and their comparison with healthy controls remain limited. Understanding the preoperative HRQoL, along with the patient’s physical and functional status, enables the development of tailored patient prehabilitation programmes. Prehabilitation includes patient education, psychological preparation, physical conditioning, and nutritional optimisation. Prehabilitation programmes have been shown to reduce postoperative pain, decrease the hospital length of stay, and improve physical function [16,17,18]. However, the evidence regarding their impact on quality of life is inconsistent [18,19].

This study was carried out to assess the HRQoL, 10MWT, and BMI in subjects awaiting arthroplasty procedure (SAP) due to end-stage OA and to compare these with age- and sex-matched healthy controls (CON). By identifying and quantifying these differences, we seek to provide a deeper understanding of how mobility impairments and reduced HRQoL contribute to the need for surgical intervention. Additionally, we explored correlations between these variables to assess how functional limitations, HRQoL, and BMI interact in individuals with end-stage OA. We hypothesised that SAP would exhibit significantly worse functional and health-related outcomes than CON. Specifically, we expected that SAP would have higher BMI, slower 10MWT speeds, and lower EQ-5D scores than CON.

## 2. Materials and Methods

### 2.1. Study Design and Participants

This cross-sectional, secondary data analysis with matched controls used a non-equivalent group design with no randomisation or assignment to the different study groups. This approach was chosen to examine naturally occurring differences between the groups, which provides real-world insights despite limitations associated with controlling confounding variables. The current study utilised data from two distinct patient cohorts established in previous projects. The SAP group was drawn from participants of the Outpatient Remobilisation after Total Knee and Hip Arthroplasty programme (AMB-REMOB project), which investigated the effectiveness of an early outpatient rehabilitation programme, incorporating underwater therapy (hydrotherapy, pressure jet massage) and electrotherapy, for patients undergoing hip or knee total joint replacement [20]. The CON group of healthy older adults was derived from the Centre of Active Ageing—Long-life Endurance Exercise and Healthy Ageing project (CAA project), which investigated the influence of endurance exercise on healthy aging by stabilizing the circadian system and improving physical fitness, body composition, bone density, and metabolic health, while also addressing its potential risks, to support policy making and promote physical activity in aging populations [21]. Both projects were carried out as part of the cross-border cooperation programme Interreg V-A Slovakia–Austria.

**Figure 1:**
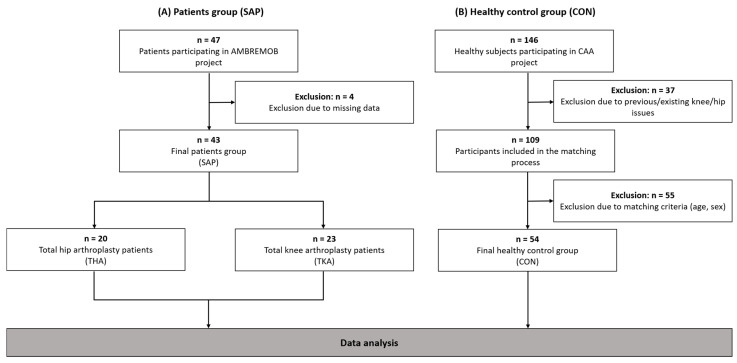
Flowchart (**A**) subjects awaiting arthroplasty procedure (SAP); (**B**) healthy control group (CON).

The AMB-REMOB project involved 173 patients who had been scheduled for TKA or THA at the orthopaedic department at the Penzing site of the Ottakring Clinic, Vienna Health Association, Austria, due to osteoarthritis between 1 April 2022 and 31 January 2023. Patients were contacted using letter-based communication, and 76 patients actively responded to the invitation by telephone or email. Following a rigorous screening process, 29 individuals were deemed ineligible based on pre-defined inclusion and exclusion criteria. Due to missing data, four individuals were excluded. The final patient’s group (SAP) for data analysis consisted of 43 patients. The SAP was divided into two subgroups: TKA (*n* = 23) and THA (*n* = 20) (Figure 1A).

The inclusion criteria for the AMB-REMOB project specified that participants were aged between 55 and 80 years and scheduled to undergo unilateral TKA or THA due to OA. Eligible individuals had to have a contralateral lower extremity with no or minimal symptoms, defined by the absence of effusion, no load-limiting pain, and a pain score of less than 5. Exclusion criteria included prior TKA or THA, a BMI exceeding 40, recent leg vein thrombosis (within three weeks), significant joint effusion, infection, and pre-existing myopathy. Additionally, neurological disorders affecting gait (e.g., polyneuropathies, Parkinson’s disease), vestibular diseases with vertigo or balance disturbances, and dementia/reduced cognitive abilities were exclusionary factors. Furthermore, the following criteria were taken into consideration: symptomatic cardiopulmonary disease within the preceding six months, uncontrolled arterial hypertension, peripheral arterial occlusive disease, malignant diseases, rheumatic conditions, and other internal contraindications. Individuals with functional impairments of the musculoskeletal system due to prior surgeries, injuries, or degenerative diseases were excluded from the study.

Initial recruitment for the CAA project was conducted from 1 November 2020 to 30 September 2022 and targeted healthy older adults aged 60 years and older, without medical conditions affecting gait or physical activity. Multiple channels of promotion were employed, including the use of the project website, placement of advertisements in print media, distribution of flyers at rehabilitation institutes, and the distribution of flyers throughout the city of St. Pölten. A total of 146 participants were enrolled during the specified period. In order to ensure comparability within the SAP, the participants were matched based on age and sex. The process of age and sex matching was executed by randomly selecting individuals from the CON group (109 participants), who were included in the matching process. This was done to ensure that the mean age and its associated standard deviation were as closely aligned as possible with those of the SAP group. This approach was adopted to ensure comparability between the two groups, while accounting for differences in their broader inclusion criteria. This resulted in a final healthy CON group of 54 participants (Figure 1B). Table 1 presents the demographic data for both groups.

The measurements for SAP were carried out at the Institute for Physical Medicine at the Wiener Gesundheitsverbund Klinik Ottakring in Vienna, Austria, as part of the AMB-REMOB project. The measurements for CON were conducted at the Faculty of Physical Education and Sport, Comenius University in Bratislava, Slovakia, as part of the CAA project. The data for both groups were collected at a single time point (baseline only), without any subsequent intervention. No financial compensation was provided to study participants, and no activities were employed to enhance compliance or adherence. The objective of both projects was to enhance the quality of life.

### 2.2. Ethical Approval

The AMB-REMOB project received ethical approval from the Ethics Committee of the City of Vienna (EK-21-255-102) and is registered in the German Register of Clinical Studies (DRKS00028152; UTN: U1111-1275-5181). The CAA project was approved by the Ethical Committee of the University Hospital Bratislava—Hospital of Ladislav Dérer (approval number 31/2020) and is registered at ClinicalTrials.gov (NCT05053282). Both studies adhere to the principles outlined in the Declaration of Helsinki. Prior to taking measurements, all participants gave their written informed consent.

### 2.3. Outcome Measures

HRQoL was assessed using the EQ-5D-5L questionnaire. This is a widely utilised instrument in health research that facilitates a preference-based assessment of health status. It comprises five dimensions: mobility, self-care, usual activities, pain/discomfort, and anxiety/depression. The EQ-5D-5L visual analogue scale (EQ VAS) was used to quantify the self-rated health status on a scale ranging from 0 to 100, while the EQ-5D index condensed all dimensions into a single value between 0 and 1 [14]. Extensive research has affirmed the tool’s effectiveness and appropriateness in evaluating health-related quality of life in patients undergoing arthroplasty or managing osteoarthritis [22]. BMI was calculated based on height and weight measurements using the following formula: $\mathrm{BMI}=\frac{\mathrm{weight}\;\mathrm{in}\;\mathrm{kg}}{{\mathrm{height}}^{2}\;\mathrm{in}\;m}$. The 10MWT was used to evaluate the participants’ walking speed over a 10-m distance, offering insights into their gait, vestibular function, and functional mobility. In this study, we followed the measurement protocol outlined by Hollman et al. [23]. Timing for the 10MWT was conducted for both the subjects’ comfortable/normal, self-paced walking speed and their fast-paced walking speed. Prior to the normal-pace trial, the participants were instructed to ‘walk with your preferred pace of walking as you would on the street not being in a hurry’. Prior to the fast-paced trial, participants were instructed to ‘walk at the fastest speed possible without running’. Each condition—normal and fast—consisted of three trials. Walking speed was determined by dividing the 10-m distance by the average time(s) required to cover this distance for each condition. Participants were permitted to use walking aides if necessary. The test demonstrates excellent test-retest reliability in patients undergoing TKA and THA [12]. The assessments were conducted over a period of approximately 30 min. All measurements were conducted by trained kinesiologists and physiotherapists in accordance with established protocols.

### 2.4. Data Analysis

First, descriptive statistics (mean, standard deviation, standard error of the mean, percentage, and percentiles) were calculated. In addition, the number and percentage of patients for each level (1–5) of each dimension of the EQ-5D-5L were reported, along with a summarised category of patients reporting the occurrence of any problem (summarizing levels 2–5). By collapsing levels together to create two categories, i.e., no problems (level 1) and any level of problems (levels 2–5), the study provides a clear understanding of the prevalence and nature of health issues experienced by each sample population. Cumulative frequencies were calculated for ordinal data [24]. The normality of the data distribution was assessed by examining the skewness, kurtosis, and by applying the Shapiro–Wilk test [25]. The difference between the groups for normally distributed data was calculated using a two-sided *t*-test for independent samples with associated 95% confidence intervals. The effect size was interpreted with Cohen’s d as follows: 0.2 (small), 0.5 (medium), and 0.8 (large) [26]. As a consequence of the matching process of SAP, stratification or analysis according to age was not conducted. The chi-square test was used to determine the differences in dimensions of HRQoL (mobility, self-care, usual activities, pain/discomfort, anxiety/depression) as well as in BMI categories between SAP and CON. A non-parametric test, the Mann–Whitney *U* test, was used for EQ-5D index and EQ VAS scores, where the effect size ‘r’ was interpreted as follows: 0.3 (small effect), 0.3–0.5 (medium effect), and >0.5 (large effect) [27]. The Spearman rank correlation coefficient test was used to determine the correlation between all dimensions of HRQoL, the results of the 10MWT, and BMI in the SAP. The correlation was interpreted as follows: 0.00–0.30 (negligible), 0.30–0.50 (low), 0.50–0.70 (moderate), 0.70–0.90 (high), and 0.90–1.00 (very high) [28]. The significance level was set at α < 0.05. In order to ensure the accuracy and consistency of the data set, any participants with incomplete or missing data were excluded prior to the finalisation of the cohort (Figure 1). Microsoft Excel for Mac version 16.77, 2023 (Microsoft, Redmond, WA, USA) and IBM SPSS Statistics 26.0.0.0 (IBM, Armonk, NY, USA) were used to analyse the data.

### 2.5. Sample Size

In order to statistically validate a large effect (*d* = 0.8) that is valid in the population via the mean values of two independent samples with a probability of error of alpha = 0.05 and a power (1 − β) of 80%, a total of *n* = 26 test subjects per sample are required [29]. A single-factor analysis of variance across three groups (*df* = 2), which exhibit moderate overall overlap (*f* = 0.25), necessitates 27 test subjects per group or 81 test subjects in total to statistically validate the differences with alpha = 0.05.

## 3. Results

The average BMI for SAP, both THA and TKA (*n* = 43), is 30.0 ± 6.1 kg/m^2^, while CON has an average BMI of 27.8 ± 3.9 kg/m^2^. THA patients (*n* = 20) have a lower average BMI of 28.8 ± 6.2 kg/m^2^ than TKA patients (*n* = 23) with 30.9 ± 5.9 kg/m^2^. The independent samples *t*-test revealed no statistically significant difference in height (*t* = 0.593; *p* = 0.554) between the SAP and CON. However, comparing the weight (*t* = 2.436; *p* = 0.017; *d* = 0.247; 95%CI = 1.324, 12.981) and BMI (*t* = 2.127; *p* = 0.037; *d* = 0.585; 95%CI = 0.140, 4.401) results shows a significant difference between these two groups.

Based on the BMI categories (underweight ≤ 18.5; normal weight = 18.5–24.9; overweight = 25–29.9; obesity = BMI of ≥30) [30], neither group included an underweight participant. About one-quarter (27.3%) of CON and only 14% of SAP had a normal weight (X^2^(19, *n* = 20) = 20, *p* = 0.395). The majority in both groups were overweight; controls, 43.6%; SAP, 46.5% (X^2^(39, *n* = 43) = 39, *p* = 0.304). In the CON group, 29.1% of the individuals were obese, compared to 39.5% in the SAP (X^2^(29, *n* = 32) = 29.3, *p* = 0.500).

**Figure 2:**
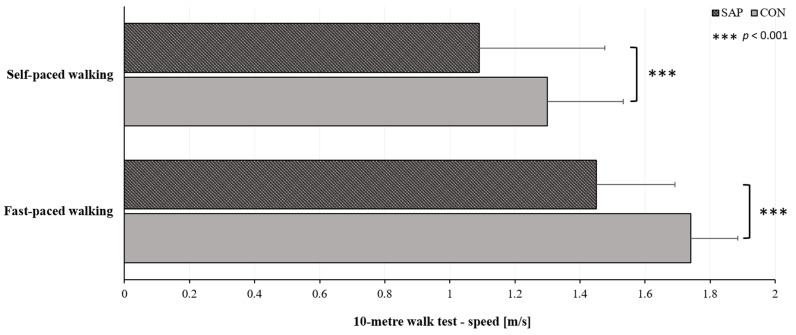
Results of speed for the 10-m walk test (self-paced and fast-paced). SAP, subjects awaiting arthroplasty procedure; CON, control group.

The results for the self- and fast-paced gait speed are shown in Figure 2. A statistically significant difference is observed between SAP and CON group when comparing self-paced speed (U = 581.0; *p* < 0.001; *r* = 0.50) as well as fast-paced speed (U = 658.0; *p* < 0.001; *r* = 0.43).

The results for HRQoL, with the number and proportion of patients reporting each level (1–5) and any problems (summarised for levels 2–5) for each dimension of the EQ-5D-5L, are presented in Table 2a,b. For all dimensions of HRQoL (mobility, self-care, usual activities, pain/discomfort, anxiety/depression), the EQ5D index, and self-rated health (EQ VAS score), a statistically significant difference is observed between the SAP and CON groups. The largest difference was found in the dimension of self-care, where 25.6% of SAP reported problems, while none of controls had any. Additionally, 65.1% of SAP reported experiencing problems regarding their ability to perform usual daily activities. Only 3.6% of participants in the CON group reported problems in this area.

**Figure 3:**
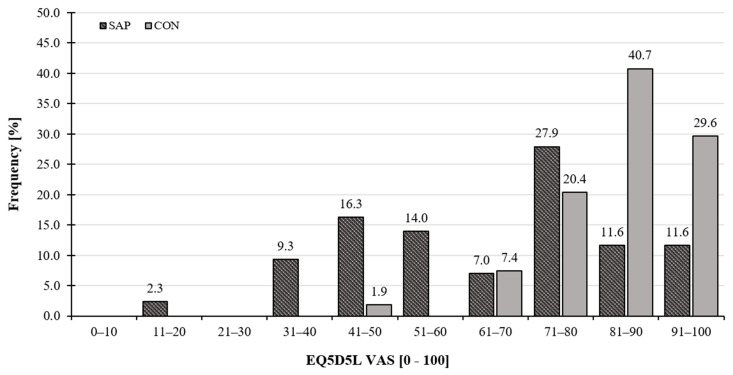
Graphical display of EQ VAS scores for SAP and CON. SAP, subjects awaiting arthroplasty procedure group; CON, control group.

The shape, central tendency of distribution, and digit preference of self-rated health (EQ VAS score) based on frequency are shown in Figure 3 for SAP and CON. Most participants (98.1%) from the CON group rated their health as >50 according to the EQ VAS score, with 11.1% even rating their health as ‘the best imaginable’ (EQ VAS score = 100). On the other hand, 27.9% of the SAP rated their state of health as ≤50, with only 2.3% rating it as ‘the best imaginable’.

As presented in Table 3, the Spearman’s correlation shows a significant correlation between dimensions of HRQoL and both 10MWT and BMI, as well as a correlation between BMI and 10MWT in the SAP group. Although we found statistically significant correlations, these were all low (*r_s_* = 0.31–0.48).

## 4. Discussion

In this study, the BMI, 10MWT, and HRQoL results were compared between SAP (*n* = 43) and CON (*n* = 54) of similar age (Table 1). While no significant differences in age or height were found between the two groups, several significant differences were observed for other measures. In particular, the SAP displayed higher body weight (*p* = 0.017) and BMI (*p* = 0.037) than individuals in the CON group, thereby supporting the hypothesis that SAP would have a higher BMI. This finding underscores the link between obesity and the need for joint arthroplasty, as obesity is a known risk factor for the development and progression of OA [1,2,31]. Additionally, it is thoroughly documented that a BMI greater than 30 increases the risk of medical and surgical complications in patients undergoing total joint arthroplasty [11]. In the SAP group, 39.5% had a BMI above 30 (vs. 29.1% CON). Based on these findings and previous research results highlighting the increased risk of complications in this SAP subgroup, we recommend that prehabilitation programmes be designed to improve the overall lifestyle (diet, physical activity) of such patients to improve their surgical outcomes after TKA or THA.

As the 10MWT has proved highly reliable in patients undergoing TKA or THA [12], we used it to demonstrate differences in locomotor function between SAP and CON. The findings support our hypothesis that SAP have slower walking speeds. In addition to having higher BMI, SAP had statistically significantly lower 10MWT speeds (*p* < 0.001) than CON (Figure 2). However, when comparing our results to the findings of Palo et al. [32], we see that our SAP had faster gait speeds (self-paced, 1.09 m/s; fast-paced, 1.45 m/s) than patients with knee osteoarthritis and atraumatic knee pain (72% with knee OA; self-paced, 0.3 m/s; fast-paced, 0.4 m/s) who were 55 years or older (*n* = 1828). In another study, patients expecting TKA (*n* = 59) displayed similar results for 10MWT time at a fast pace six weeks before surgery (7.7 ± 1.8 s) [33] as our SAP (7.51 ± 2.47 s). The significant differences observed in our study between SAP and CON in terms of 10MWT speed (Figure 2) highlight the impact of OA on gait speed and physical performance. However, Storey et al. [34] concluded that functional outcomes (timed up and go test, stair climb test, 10MWT, six-minute walk test) were similar between post-TKA subjects and a control group. Our results reflect the functional limitations faced by individuals awaiting joint replacement. These findings underscore the importance of evaluating and addressing mobility limitations in prehabilitation assessments and postoperative rehabilitation programmes for TKA and THA patients.

Previous research has shown that HRQoL improves after TKA or THA, but few studies have examined HRQoL in patients awaiting these procedures or compared them with healthy individuals of similar age [35]. The current analysis found significant differences in all HRQoL domains between the SAP and CON (Table 3), with the most striking discrepancies observed in the dimensions of self-care and the ability to perform activities of daily living. Palo et al. [32] found that patients with knee OA and atraumatic knee pain had a moderately low quality of life based on SF-36 and mental component score results, which included both physical and psychological aspects. However, it is difficult to directly compared their results with ours, as the SF-36 and the mental component score were used in the study, while the EQ-5D-5L was used to assess HRQoL in the present study. The mean EQ-5D index scores of SAP (0.67 ± 0.25) were higher than those reported previously for TKA (men, 0.58 ± 0.28; women, 0.51 ± 0.29) and THA (male = 0.63 ± 0.25; female = 0.53 ± 0.28) patients before surgery [36]. This discrepancy is believed to be due to the much larger sample size in the above-mentioned study (TKA *n* = 1629, THA *n* = 2368). Bischof et al. [36] recommended using individual dimensions of the questionnaire instead of the index score, but the findings on these separate dimensions are not commonly reported for patients awaiting TKA or THA.

This study explores the relationships between HRQoL dimensions, functional mobility, and BMI in detail, shedding light on their clinical relevance in patients with advanced musculoskeletal conditions. The findings demonstrate that not all HRQoL domains are equally affected (Table 2a); mobility, usual activities, and pain emerged as the most impacted areas. This may highlight the critical role of maintaining independence and engagement in daily life both to ensure individual well-being and to achieve broader public health outcomes. It is noteworthy that mobility and usual activities exhibited the strongest correlations with 10MWT (*r_s_* = −0.21–0.45; Table 3), underlining the significance of functional mobility as a vital indicator of overall health status and surgical necessity. Such correlations suggest that targeted clinical interventions could be particularly beneficial for patients with diminished physical function, especially in domains related to daily activities. While pain was identified as a prominent differentiator between SAP and CON (Table 2a), its correlation with the 10MWT was less strongly pronounced (*r_s_* = −0.30; Table 3). This result implies that an evaluation in participation-oriented domains, such as usual activities, provides a more robust and actionable assessment of patient needs. In addition, the correlation between BMI and mobility impairment, as demonstrated in Table 3, indicates that a higher BMI is associated with reduced walking speed and greater functional limitations. As noted in prior studies, this finding suggests that patients with a higher BMI and reduced physical fitness are more likely to seek surgical intervention earlier [37]. As demonstrated in Table 2a, SAP exhibited significantly lower HRQoL scores across multiple dimensions compared to the CON group. These findings highlight the potential impacts of obesity and mobility loss, emphasising the necessity to incorporate these factors into prehabilitation strategies. From a clinical standpoint, the integration of both subjective (PROMs) and objective (CROMs) measures may facilitate the identification of subjects awaiting arthroplasty who would most benefit from prehabilitation and timely surgical intervention. This approach has the potential to enhance patient outcomes by addressing the health domains most relevant to daily functioning.

Our findings emphasise the significant impact of OA on patients’ daily functioning and overall well-being, indicating that comprehensive support and interventions before surgery are needed to improve quality of life in this population. The prehabilitation approach, which includes body mass control, physical activity, and patient education, plays a crucial role in optimising patient outcomes prior to surgery. In turn, this leads to faster recovery, fewer postoperative complications, and potentially shorter hospital stays. However, implementing exercise programmes can be challenging for some patients, especially those with pre-existing conditions or limited functional mobility. Therefore, it is crucial to tailor prehabilitation programmes to meet the individual patient’s needs, maximise the benefits of this approach, and ultimately improve total joint arthroplasty outcomes [35,38,39].

The present study acknowledges certain limitations that may affect the generalisability of its findings. Firstly, the relatively small sample size and the combined analysis of patients awaiting TKA and THA may limit the applicability of the results to a broader population. While statistically significant correlations were observed between HRQoL dimensions, 10MWT performance, and BMI in the SAP, the overall strength of these correlations was generally weak. However, certain HRQoL dimensions, most notably the usual activities, exhibited stronger associations with BMI, suggesting that specific domains of subjective measures align more closely with objective measures. This finding indicates that these targeted domains may reflect the patient experience and disease burden more accurately. The cross-sectional design of the study does not enable us to draw causal inferences, which prevented us from determining whether impaired functional mobility or higher BMI is the cause of the observed relationships. Furthermore, relying on the 10MWT as the exclusive mobility assessment may not enable aspects such as endurance, balance, or quality of gait to be fully assessed. We recommend that further research is conducted to explore these mechanisms and assess whether the examination of specific HRQoL domains enables better predictions for functional decline and surgical decision-making. Despite the limitations mentioned above, the study provides valuable insights into the multidimensional nature of HRQoL, mobility, and BMI, highlighting the need to take a more comprehensive approach towards evaluating functional status and optimising outcomes for patients awaiting arthroplasty.

## 5. Conclusions

This study highlights the substantial impact of OA on functional mobility and HRQoL in patients awaiting TKA or THA. SAP demonstrated slower 10MWT speeds and greater impairments in HRQoL, where mobility, usual activities, and pain were identified as the most strongly affected domains. The strong association between mobility impairments and reduced HRQoL underscores how heavily OA limits daily functioning and contributes to the need for surgical intervention. While functional decline is pivotal to this process, factors such as BMI may also influence functional mobility and activity levels. The findings emphasise that functional mobility loss is not merely a symptom of OA, but rather a significant factor influencing a patient’s decision to undergo surgery. It is imperative to address functional decline prior to surgery, and the provision of early, targeted interventions is essential. A structured prehabilitation programme emphasising physical activity and patient education is recommended to enhance physical function and quality of life prior to surgery.

## Figures and Tables

**Table 1:** Demographic data.

	SAP (*n* = 43)	THA (*n* = 20)	TKA (*n* = 23)	CON (*n* = 54)
	*Mean* ± *SD*			*Mean* ± *SD*
**Age (years)**	66.7 ± 8.7	65.6 ± 9.1	67.5 ± 8.3	65.6 ± 1.5
**Weight (kg)**	84.7 ± 16.0	83.3 ± 17.7	85.6 ± 14.9	77.5 ± 13.0
**Height (cm)**	168.2 ± 9.9	170.2 ± 10.5	166.8 ± 9.4	167.0 ± 10.0
**Sex (m/f)**	17/26	9/11	8/15	21/34
**Involved side (L/R)**	20/23	9/11	10/13	NA

*n*, sample size; SAP, subjects awaiting arthroplasty procedure; THA, total hip arthroplasty; TKA, total knee arthroplasty; CON, control group; SD, standard deviation; m, male; f, female; L, left; R, right; NA, not applicable. The independent samples *t*-test revealed no statistically significant difference in age (*p* = 0.415).

**Table 2:** (**a**) Proportion of patients and controls reporting levels 1 to 5 for all EQ-5D-5L dimensions. (**b**) Assessment of EQ-5D index and EQ VAS score in SAP versus control group.

(**a**)											
**EQ-5D-5L** **Dimensions**		**EQ-5D-5L Levels** **%**					**Reported Problems** **%**		**Chi-Square Test**		
	**G**	**1**	**2**	**3**	**4**	**5**			**X^2^**	***df***	***p***
**Mobility**	SAP	14	25.6	37.2	20.9	2.3	86		61.9	4	<0.001
	CON	92.7	5.5	1.8	0	0	7.3				
**Self-care**	SAP	74.4	18.6	7	0	0	25.6		15.6	2	<0.001
	CON	100	0	0	0	0	0				
**Usual** **activities**	SAP	34.9	32.6	20.9	11.6	0	65.1		42.4	3	<0.001
	CON	96.4	1.8	1.8	0	0	3.6				
**Pain** **/discomfort**	SAP	2.3	16.3	44.2	34.9	2.3	97.7		59.3	4	<0.001
	CON	41.8	50.9	5.5	1.8	0	58.2				
**Anxiety** **/depression**	SAP	55.8	23.3	11.6	9.3	0	44.2		17.0	3	<0.001
	CON	89.1	10.9	0	0	0	10.9				
(**b**)											
		**G**	**Mean**	**SD**	**SEM**	**Median**	**Q1**	**Q3**	**Mann-Whitney *U* test**		
									**U**	***r***	***p***
**EQ-5D Index**		SAP	0.671	0.202	0.031	0.748	0.547	0.810	96.0	0.92	<0.001
		CON	0.937	0.066	0.009	0.910	0.910	1.000			
**EQ VAS score**		SAP	68.4	19.0	2.9	75	50	80	269.5	0.60	<0.001
		CON	86.9	10.0	1.4	90	80	95			

G, group; *r*, effect size; *p*, statistical significance; SAP, patient group; CON, control group; SD, standard deviation; SEM, standard error of the mean; VAS, visual analogue scale; Q1, 25th percentile; Q3, 75th percentile. X^2^, continuous probability distribution; *df*, degrees of freedom.

**Table 3:** Spearman’s correlation between different dimensions of HRQoL and both 10MWT and BMI and correlation between BMI and 10MWT in the SAP group.

EQ-5D-5L Dimension	Correlated Variables	Spearman’s Correlation	
		*r_s_*	*p*
**Mobility**	10 m normal speed	−0.34	0.026 *
	10 m fast speed	−0.21	0.174
	BMI	0.44	0.003 **
**Self-care**	10 m normal speed	−0.27	0.077
	10 m fast speed	−0.23	0.158
	BMI	0.31	0.042 *
**Usual activities**	10 m normal speed	−0.32	0.039 *
	10 m fast speed	−0.45	0.003 **
	BMI	0.34	0.026 *
**Pain/discomfort**	10 m normal speed	−0.30	0.051
	10 m fast speed	−0.26	0.098
	BMI	0.47	0.002 **
**Anxiety/depression**	10 m normal speed	−0.27	0.080
	10 m fast speed	−0.29	0.056
	BMI	0.17	0.276
**Index**	10 m normal speed	0.41	0.007 *
	10 m fast speed	0.31	0.046 *
	BMI	−0.41	0.006 *
**Self-rated health** **(EQ VAS score)**	10 m normal speed	0.23	0.142
	10 m fast speed	0.30	0.051
	BMI	−0.19	0.231
**BMI**	10 m normal speed	−0.48	0.001 **
	10 m fast speed	−0.44	0.003 **

BMI, body mass index; VAS, visual analogue scale; *r_s_*, correlation coefficient; *p*, statistical significance; * *p* < 0.05; ** *p* < 0.01.

## Data Availability

The research data supporting this publication are stored at our institutional digital data repository for published research accessible via https://creed.lbg.ac.at (accessed on 28 December 2024). The data sets analysed in this manuscript are not publicly available due to ethical and legal restrictions (data contain potentially identifying and sensitive patient information). However, pseudonymised data sets have been created for the purpose of re-use and are also accessible via creed.lbg.ac.at. Requests for access to anonymised data sets should be directed to the corresponding author [VG].
